# Thermally robust spin correlations between two ^85^Rb atoms in an optical microtrap

**DOI:** 10.1038/s41467-019-09420-6

**Published:** 2019-04-23

**Authors:** Pimonpan Sompet, Stuart S. Szigeti, Eyal Schwartz, Ashton S. Bradley, Mikkel F. Andersen

**Affiliations:** 1 The Dodd-Walls Centre for Photonic and Quantum Technologies, Department of Physics, University of Otago, Dunedin, New Zealand; 2 Department of Quantum Science, Research School of Physics and Engineering, The Australian National University, Canberra, ACT 2601 Australia; 3 Present Address: Max-Planck-Institut für Quantenoptik, 85748 Garching, Germany

**Keywords:** Atomic and molecular collision processes, Ultracold gases, Quantum physics

## Abstract

The complex collisional properties of atoms fundamentally limit investigations into a range of processes in many-atom ensembles. In contrast, the bottom-up assembly of few- and many-body systems from individual atoms offers a controlled approach to isolating and studying such collisional processes. Here, we use optical tweezers to individually assemble pairs of trapped ^85^Rb atoms, and study the spin dynamics of the two-body system in a thermal state. The spin-2 atoms show strong pair correlation between magnetic sublevels on timescales exceeding one second, with measured relative number fluctuations 11.9 ± 0.3 dB below quantum shot noise, limited only by detection efficiency. Spin populations display relaxation dynamics consistent with simulations and theoretical predictions for ^85^Rb spin interactions, and contrary to the coherent spin waves witnessed in finite-temperature many-body experiments and zero-temperature two-body experiments. Our experimental approach offers a versatile platform for studying two-body quantum dynamics and may provide a route to thermally robust entanglement generation.

## Abstract (web summary)

Spin-changing atomic collisions are important for thermally robust entanglement generation with applications in quantum information. Here the authors demonstrate record high spin state correlations and long spin relaxation times in the collision of two Rb atoms at relatively warm temperatures.

## Introduction

When two atoms collide their interaction is complex, leading to a wide range of possible outcomes. The result of the collision strongly depends upon experimental parameters such as the internal atomic states, the collisional energy, and external electromagnetic fields^1^. Modern atomic physics experiments exploit the richness of these atomic interactions to engineer systems for a remarkable variety of purposes, including quantum information processing^2^ and quantum simulation^3,4^. A wealth of physical phenomena have been simulated with cold atoms, such as black holes^5^ and superconductivity^6^. Of particular importance to atomic simulations of quantum magnetism is the local spin-changing interaction between atoms in their groundstate manifold^7,8^.

In many-body experiments, spin-changing collisions lead to coherent spin waves in both quantum-degenerate and thermal atomic samples^9–15^. These spin waves manifest as time-dependent populations of the atoms’ magnetic sublevels. Spin-changing collisions have additionally been used to generate quantum-entangled samples of ten thousand atoms^16^. Such entanglement has enabled sub-shot-noise phase measurements with matter-wave interferometers^17,18^ and has recently allowed fundamental studies of Einstein-Podolsky-Rosen (EPR) steering with atomic clouds^19–21^.

Unfortunately, detailed investigations of spin-changing collisions in many-atom experiments is challenging, due to undesirable processes including three-body loss^22,23^. The superfluid to Mott insulator transition provides one means of separating atomic pairs for ‘clean’ studies of spin-changing collisions^24,25^. However, this is limited to atomic species with collisional properties suitable for Bose condensing and subsequent manipulation. Consequently, experimental tests of the predicted ^85^Rb spin-dependent interaction strengths^26^ have remained elusive, and in general atomic species with negative background scattering lengths suffer unique experimental difficulties in the many-body regime^8^.

A more versatile, bottom-up approach^27,28^ is to prepare and manipulate individual atomic pairs via optical tweezers, enabling studies of interactions between any combination of atoms that can be laser-cooled. However, to date such studies have been restricted to inelastic interactions that cause atom loss^29–32^ and interactions where no overall population dynamics occur^33^.

Here, we study spin-changing collisions between individual pairs of ^85^Rb atoms prepared in an optical tweezer, and observe the collision-driven population dynamics of the magnetic sub-states in the groundstate manifold. We observe record-high suppression of relative number fluctuations and find that a bias magnetic field strongly affects the dynamics. The observed crossover from fast relaxation dynamics at low-bias field to slow, field-independent relaxation dynamics at the higher fields is captured by simulations based upon a simplified atom–atom interaction. However, for high magnetic fields the very large system of coupled modes involved at the experimental temperature prohibits quantitative first-principles modelling of the observed slow relaxation of spin-state populations. Nonetheless, in this regime the experimental data is well-fitted using incoherent rate equations with a single-parameter fit, where the relative coupling rates between different spin states are deduced from the theoretically predicted ^85^Rb spin-dependent interaction strengths^26^.

## Results

### Experimental sequence

**Fig. 1:**
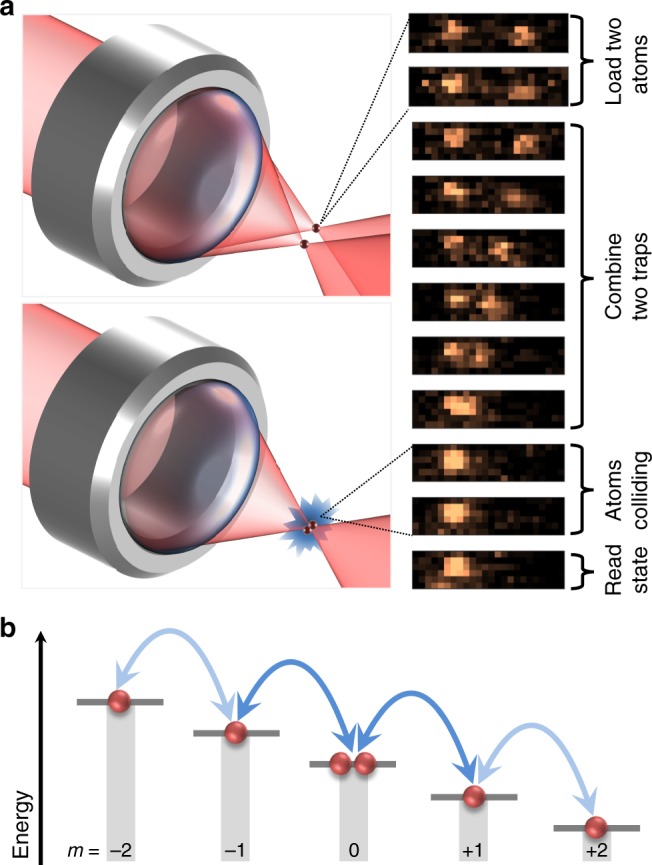
Experimental schematics. **a** (Left) Two optical tweezers are formed using the high-numerical-aperture lens. By reducing the separation between the tweezers and then turning one of the tweezers off, both atoms are transferred into the same optical tweezer, allowing atomic collisions to occur. (Right) Superimposed fluorescent images of the same two atoms showing their relative positions for different experimental stages. After combining the two traps, the individual atomic positions can no longer be resolved. **b** Spin-changing collisions: Two atoms initially in |0, 0〉 can only couple to $\hat S\left| {1, - 1} \right\rangle$ (dark arrows) and then to $\hat S\left| {2, - 2} \right\rangle$ (light arrows), where the symmetrization operator $\hat S$ is defined in the main text

Our experiments employ two ^85^Rb atoms, initially loaded into two separated optical tweezers^29,34,35^, and prepared in the *f* = 2, *m* = 0 groundstate (see Fig. 1a). The two optical tweezers are then merged, leaving the pair in a single tweezer. The magnetic bias field is set to the desired value and the two atoms are held within the single tweezer for a specified duration, which we hereafter refer to as the collision time. After a given collision time, the atomic *m*-states (denoted |*m*〉) are measured by ejecting atoms in a particular |*m*〉 and measuring the remaining atom number (see Methods for details and experimental parameters).

### Model

Once in the same optical tweezer, the two atoms interact via interaction Hamiltonian $\hat H_{\mathrm{s}}$, which depends on the pair’s relative position and spin state. Approximating the optical tweezer as an *m*-independent harmonic potential separates the centre-of-mass and relative motions of the two atoms, decoupling the internal spin and centre-of-mass dynamics, and permitting a simplified description via Hamiltonian^24,25,36,37^

1 $$\hat H = \frac{{{\widehat{\mathbf{p}}}^2}}{{2\mu }} + \mathop {\sum}\limits_{j = x,y,z} \frac{1}{2}\mu \omega _j^2\hat r_j^2 + \mathop {\sum}\limits_{i = 1,2} \hat H_{{\mathrm{Z}},i} + \hat H_{\mathrm{s}},$$

where $\widehat {\mathbf{r}} = (\hat r_x,\hat r_y,\hat r_z)$ and $\widehat {\mathbf{p}}$ are relative position and momentum operators, respectively, *μ* the reduced mass, *ω*_*j*_ the atomic oscillation frequency in the *j*^th^ dimension, and $\hat H_{Z,i}$ the Zeeman shift for the *i*^th^ atom. Our experiments use thermal atoms with *k*_B_*T* much larger than ℏ*ω*_*j*_, Zeeman energies, and atomic interaction energies.

Under suitable approximations, $\hat H_{\mathrm{s}}$ conserves total magnetization^24,25,38^ and two atoms initially prepared in *m*_1_ = *m*_2_ = 0 are restricted to bosonic symmetrized states with *m*_1_ = −*m*_2_: |0, 0〉 = |0〉_1_ $\otimes$ |0〉_2_, $\hat S\left| {1, - 1} \right\rangle = \frac{1}{{\sqrt 2 }}\left( {\left| 1 \right\rangle _1 \otimes \left| { - 1} \right\rangle _2 + \left| { - 1} \right\rangle _1 \otimes \left| 1 \right\rangle _2} \right)$, and $\hat S\left| {2, - 2} \right\rangle = \frac{1}{{\sqrt 2 }}\left( {\left| 2 \right\rangle _1 \otimes \left| { - 2} \right\rangle _2 + \left| { - 2} \right\rangle _1 \otimes \left| 2 \right\rangle _2} \right)$ (see Fig. 1b). Here $\hat S$ denotes the symmetrization operator, |*m*_1_, *m*_2_〉 the unsymmetrized two-particle spin states, and subscripts 1 and 2 denote the two atoms.

### Spin correlations

**Fig. 2:**
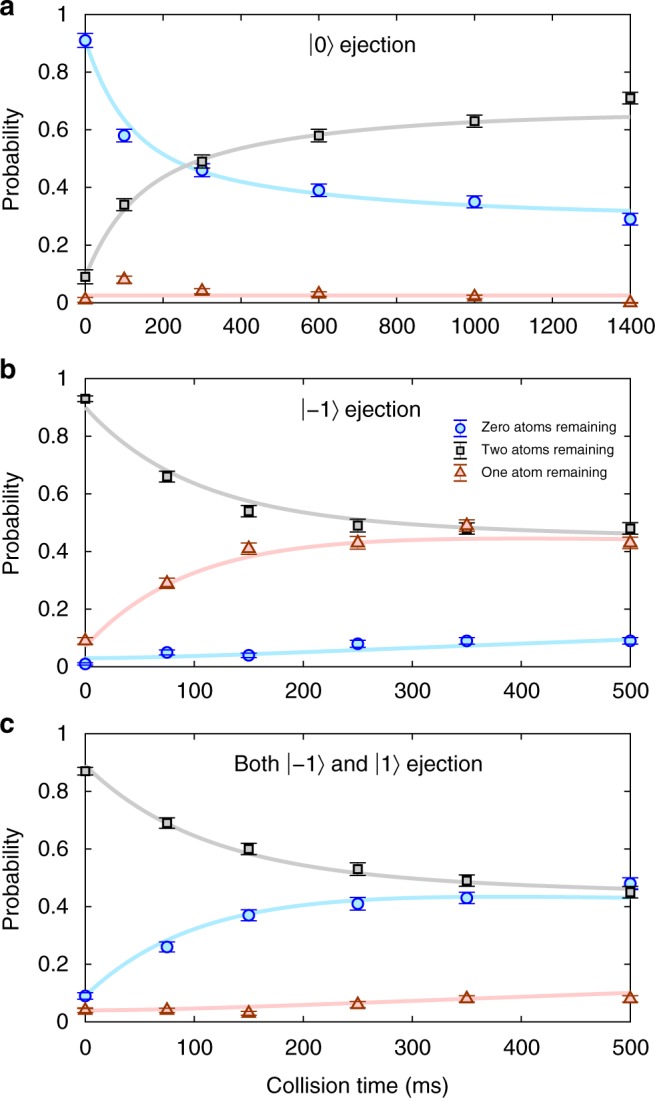
*m*-state correlation results. Probability that zero, one, or two atoms remain in the optical tweezer after a given collision time. **a** When atoms in |0〉 are expelled (immediately after a given collision time), the probability that both atoms were in |0〉 (and therefore ejected) decreases, while the probability that both atoms remain correspondingly increases. **b**, Expelling atoms solely from |−1〉 gives only single-atom loss events. **c**, Expelling atoms from both |−1〉 and |1〉 gives only pair loss, in strong contrast to the result in **b**. In all cases and throughout the collision time, the bias magnetic field was 8.5 Gauss. Error bars in all panels denote the standard error of the mean. The solid curves are fits to the data included to guide the eye. Similar data that demonstrates correlations between |−2〉 and |+2〉 is shown in Supplementary Fig. 1. Source data are provided as a Source Data file

By measuring magnetic sublevels of the atomic pair for different collision times, we confirm that the spin dynamics is governed by the simple model of spin-changing collisions depicted in Fig. 1b, which yields strong correlations between the *m*-states in a given pair. This requires the three measurement series summarized in Fig. 2. A particular |*m*〉 is detected by ejecting atoms in this state. In Fig. 2a we expel atoms in |0〉 after a given collision time. The probability that both atoms are in |0〉 (i.e., no remaining atoms) decays with increasing collision time, while the probability that both atoms remain grows correspondingly. The probability of observing one remaining atom is always negligible, implying that collisions cause both atoms to leave |0〉 simultaneously. In Fig. 2b we start with both atoms in |0〉 but eject atoms in |−1〉. The probability that one atom is in |−1〉 grows with collision time, but both are never |−1〉, since the probability that both atoms are ejected is effectively zero. In Fig. 2c we eject atoms in both |−1〉 and |1〉. This ejects both atoms, or none. Combining this with Fig. 2b, we conclude that when one atom is in |−1〉, the other is in |1〉. The populations of |−1〉 and |1〉 are therefore almost perfectly correlated. Similar data for |±2〉 shows these populations are also correlated (see Supplementary Note 1). The lasting pair correlation on timescales exceeding one second is facilitated by having individual atomic pairs. In contrast, in many-body experiments with spin-2 atoms, subsequent spin-changing collisions would likely deteriorate such strong pair correlations.

We quantify the pair correlation with the relative number squeezing, *ζ*^ 2^ (see Methods). Without correcting for finite detection efficiency, it is 11.9 ± 0.3 dB below quantum shot noise (QSN) for the |±1〉 populations. Since our atomic-pair ensemble is thermal, this large pair correlation is thermally robust. *ζ*^2^ is limited solely by our detection efficiency (see Methods); improved detection efficiency could reduce *ζ*^2^ by a further order of magnitude. For many-body systems, the highest reported relative number squeezing via spin-changing collisions is 11.4 dB below QSN (12.4 dB after correcting for detection inefficiency)^39^.

### Magnetic field dependence

**Fig. 3:**
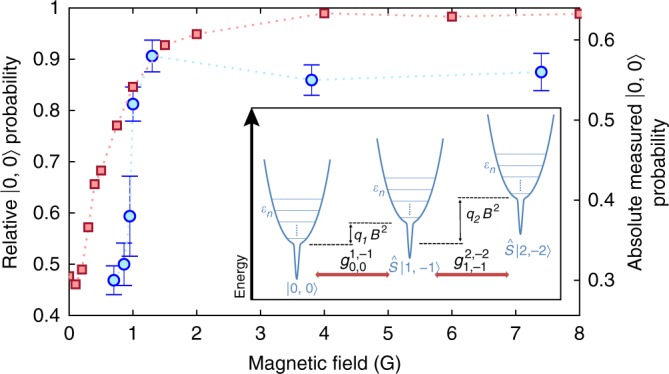
Effect of bias magnetic field. The left axis represents the |0, 0〉 population at 40 ms of collision time relative to the |0, 0〉 population at *t* = 0. Both the experimental (blue circles) and simulation (red squares) results are plotted as a function of the magnetic field. Error bars in the experimental data denote the standard error of the mean. Although the |0, 0〉 population of the simulation at *t* = 0 is set to 1, in the experiment the population dynamics during the magnetic-field ramp leave a |0, 0〉 population of about 0.64 at *t* = 0. The right axis is the actual scale of the experimentally measured |0, 0〉 population. The inset schematically shows the energy-level picture of the system. Atomic pairs in a given spin mode have accessible energies $\varepsilon _n = \hbar \omega _x(n_x + {\textstyle{1 \over 2}}) + \hbar \omega _y(n_y + {\textstyle{1 \over 2}}) + \hbar \omega _z(n_z + {\textstyle{1 \over 2}})$, constrained by $( - 1)^{n_x + n_y + n_z} = 1$. A magnetic field of strength *B* shifts the energy levels of modes $\hat S|1, - 1\rangle$ and $\hat S|2, - 2\rangle$ by $q_1B^2$ and $q_2B^2$, respectively, due to the quadratic Zeeman effect. Spin-changing collisions couple these energy levels, with coupling strengths $g_{m_1,m_2}^{m_{1}^{\prime},m_{2}^{\prime}}$. See Methods for further details. Source data are provided as a Source Data file

The bias magnetic field affects the spin dynamics through $\mathop {\sum}\nolimits_i {\hat H_{{\mathrm{Z}},i}}$. Since our model conserves total magnetization, the first-order Zeeman contributions cancel for the accessible two-body states, so $\mathop {\sum}\nolimits_i {\hat H_{{\mathrm{Z}},i}}$ only contributes via second-order terms. We investigate how $\mathop {\sum}\nolimits_i {\hat H_{{\mathrm{Z}},i}}$ affects the spin dynamics by measuring the |0, 0〉 population after a 40 ms collision time for different bias fields (Fig. 3). At low biases, the dynamics are highly magnetic-field dependent, whereas for higher biases the dynamics are effectively magnetic-field independent. Here typical thermal energies are much larger than second-order Zeeman energies for all biases investigated. The atom pairs, therefore, have sufficient thermal energy to overcome the Zeeman shift when undergoing spin-changing collisions, so, in contrast to ultracold samples, the dynamics should not necessarily quench at high biases.

To understand the spin evolution, we simulated the dynamics governed by Eq. (1) with a simplified interaction $\hat H_{\mathrm{s}} = V\left( {\hat {\mathbf{r}}} \right) \times \mathop {\sum}\nolimits_{m_{1},m_{2},m_{1}{\hskip -3pt \prime} ,m_{2}{\hskip -3pt \prime} } {g_{m_{1},m_{2}}^{m_{1}{\hskip -3pt \prime} ,m_{2}{\hskip -3pt \prime} }} \left| {m_{1}{\hskip -3pt \prime} ,m_{2}{\hskip -3pt \prime} } \right\rangle \langle m_{1},m_{2}|$, where $g_{m_1,m_2}^{m_1{\hskip -3pt \prime} ,m_2{\hskip -3pt \prime} }$ are determined from predicted spin-dependent *s*-wave scattering lengths^26^ and $V\left( {\hat {\mathbf{r}}} \right)$ is a Gaussian with width chosen to reproduce the total free-space *s*-wave collision cross section (see Methods). A Gaussian pseudopotential moderates problems that afflict zero-length interaction potentials in tight traps^36,37^, while still avoiding the complexity of a more complete $\hat H_{\mathrm{s}}$.

The simulation was conducted by averaging over a thermal ensemble of initial states evolved using Eq. (1). The initial states were relative-motion eigenstates of $\hat {\mathbf{p}}^2/(2\mu ) + \mathop {\sum}\nolimits_j {{\textstyle{1 \over 2}}} \mu \omega _j^2\hat r_j^2$ with two-particle spin state |0, 0〉. Due to the prohibitively large Hilbert space required at the experimental temperature, simulations were restricted to a lower temperature of 8.8 μK. All simulations at this temperature were performed on a finite basis of 16,996 relative-motional modes.

The simulation qualitatively captures the spin dynamics (Fig. 3). We observe a crossover from fast dynamics at low magnetic-field strengths to slow dynamics at high fields. $\hat H$ couples the three allowed spin modes, |0, 0〉, $\hat S\left| {1, - 1} \right\rangle$, and $\hat S\left| {2, - 2} \right\rangle$ (inset, Fig. 3). When the pair is in a particular spin mode, it behaves as an effective single particle within a harmonic trap with the interaction potential placed at the trap centre. At low magnetic fields, $\mathop {\sum}\nolimits_i {\hat H_{Z,i}}$ is negligible, so any relative-motion eigenstate with a particular spin mode (e.g., |0, 0〉) is approximately degenerate to relative-motion eigenstates in other spin modes (e.g., $\hat S\left| {1, - 1} \right\rangle$, and/or $\hat S\left| {2, - 2} \right\rangle$); the degeneracy is only lifted by the atom-atom interaction’s spin-state dependence. The resulting resonant coupling efficiently transfers the population between spin modes at low magnetic fields. In contrast, at high fields this degeneracy is lifted, the majority of initially occupied states have no near-resonant coupling to other spin modes, leaving only off-resonant coupling, and the dynamics largely cease.

### The high magnetic field regime

**Fig. 4:**
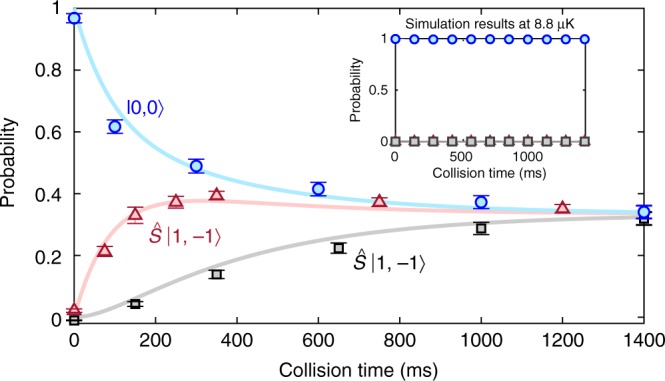
Spin population dynamics at high bias field. The populations of the two-atom states are plotted as a function of collision time with error bars denoting the standard error of the mean. The solid curves are a fit of the measured data with spin-changing rate equations, while the ratio of the rates between $\left| {0,0} \right\rangle \, \rightleftharpoons \,\hat S\left| {1, - 1} \right\rangle$ and $\hat S\left| {1, - 1} \right\rangle \, \rightleftharpoons \, \hat S\left| {2, - 2} \right\rangle$ is determined from the theoretically predicted spin-dependent interaction strengths. The bias field was 8.5 Gauss for all collision times. The inset illustrates that the simplified theoretical model used for our simulations fails to capture the long-time relaxation dynamics in the high magnetic field regime. Source data are provided as a Source Data file

Figure 3 shows a quantitative difference between simulation and experiment. In the high bias, magnetic-field-independent regime, the simulation gives |0, 0〉 population at *t* = 40 ms close to the *t* = 0 population, while in the experiment it is lower. Figure 4 demonstrates the cause of this difference. The experiment shows slow relaxation to equal populations of the three spin modes, while the simulation dynamics are quenched (no spin-changing collisions). Here the equal population is not complete thermalization within states that conserve total magnetization; since atoms with different internal states can be considered distinguishable, the thermalized populations with *m* = ±1 and *m* = ±2 would be twice that of |0, 0〉.

Generally, a priori calculations of thermal decoherence in colliding atomic ensembles pose a challenge for theory, often necessitating phenomenological rate-equation approaches to account for dissipation^40–43^. In our system, several effects that are not included in the simulations might explain the dynamics in Fig. 4. Magnetic field noise might affect the dynamics or slight polarization pollution of the optical tweezer light could give a slightly *m*-dependent trap, the latter invalidating our separation of the pair’s centre-of-mass and relative coordinates. The non-paraxial nature of the optical tweezers inevitably introduces a spatially varying polarization that can be described as a fictitious magnetic field gradient^44^. We suppress the effect of this by having the bias magnetic field perpendicular to the fictitious field. A more realistic atom-atom interaction $\hat H_{\mathrm{s}}$ may also introduce new collisional timescales not captured by our simulations’ simplified interaction. Finally, the five-fold temperature difference between our simulations’ practical limit and the experimental temperature could play a role. However, this appears an unlikely explanation, as the simulation does not reveal long-time dynamics for any of the temperatures we investigated. Note that refs. ^24,25^ also included fitted relaxation rates with timescales similar to what we observe in Fig. 4, and this was needed in order to match their experimental observations to theoretical predictions.

Figure 4’s data is well-modelled using rate equations (see Methods). Incoherent transition rates likely depend on the cross section for the process, which is proportional to the squared magnitude of the coupling matrix elements. These are determined from theoretically predicted ^85^Rb spin-dependent interaction strengths^26^. Based on this, the ratio of the rates between $\left| {0,0} \right\rangle \, \rightleftharpoons \, \hat S\left| {1, - 1} \right\rangle$ and $\hat S\left| {1, - 1} \right\rangle \, \rightleftharpoons \, \hat S\left| {2, - 2} \right\rangle$ is 2.34, while the rate between $\left| {0,0} \right\rangle \, \rightleftharpoons \, \hat S\left| {2, - 2} \right\rangle$ is negligible. Fitting using a single overall rate as the fitting parameter matches the data very well (Fig. 4), indicating that the ratios between the rates is determined by the ratios between the collisional cross sections. Figure 3 therefore displays a crossover from a resonant coupling regime at low magnetic fields to a regime at high fields where the collision dynamics do not depend upon the energy difference between different spin states. Although an incoherent rate equation model gives a good fit to the collisional dynamics in the high magnetic field regime, it is incapable of providing an explanation of the magnetic-field dependence of the relaxation timescale in the low bias regime. The coupling matrix elements are independent of bias magnetic fields in the range we consider, and models that ignore quantization of the motional states do not capture the change in resonance condition that changing the bias field gives rise to.

### The low magnetic field regime

**Fig. 5:**
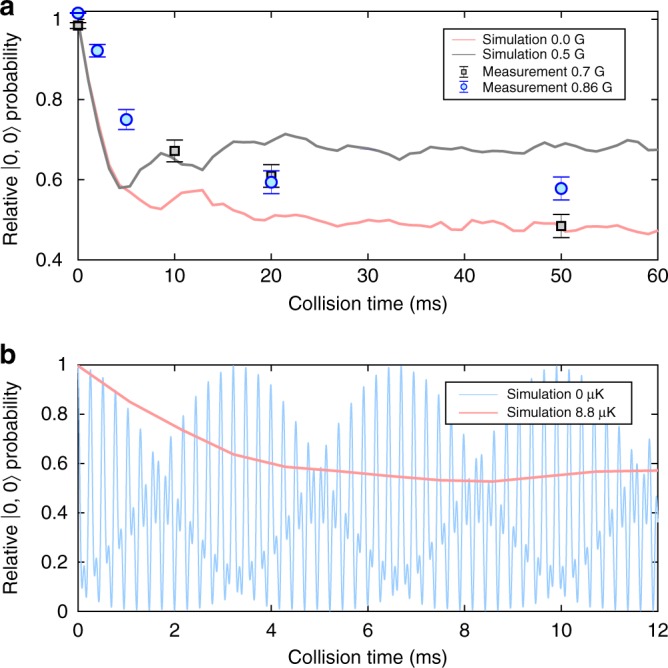
Spin population dynamics at low bias field. **a** Comparison between simulated and measured relative populations of |0, 0〉 at low magnetic bias fields. Error bars in the experimental data denote the standard error of the mean. **b** Simulation of the |0, 0〉 population as a function of collision time at two different temperatures and zero magnetic bias field. The initial relative motional state for the zero temperature simulation was the interacting groundstate of the relative motional Hamiltonian $\langle 0,0|\hat H|0,0\rangle$. The zero temperature simulation was performed on a truncated basis of 316 relative motional modes. Source data are provided as a Source Data file

Figure 5a shows the measured and simulated |0, 0〉 populations as a function of collision time in the low bias-field regime. Both experimental data and simulation display spin relaxation dynamics. This is contrary to finite-temperature many-body experiments^11,14^ which exhibit high-contrast coherent oscillations between spin modes. The observed relaxation dynamics of the two-atom system can be understood from the form of the coupling matrix elements (that include the elements of **T**, see Methods) that couple the different spin and relative motional states. Coupling between any two relative motional eigenstates is strongly dependent upon the relative motional energies of these two states. They have a tendency to decrease as the relative motional energy increases, reflecting that the overall interaction decreases with increasing energy. Consequently, the timescale of the dynamics depends upon the initial relative motional state. Although each initial atom-pair state displays coherent oscillations, averaging over a thermal distribution of these initial states, therefore, washes out the oscillations, resulting in relaxation dynamics. This is illustrated in Fig. 5b, which shows a simulation of the |0, 0〉 population for two different temperatures. At zero temperature, where only the relative motional groundstate is initially populated, we observe coherent oscillations similar to those in ref. ^24^, while at 8.8 μK we see relaxation. Finally, since the coupling matrix elements decrease with increasing motional energy we also expect the lower temperature simulation to display faster dynamics than the experiment, consistent with Fig. 5a.

## Discussion

Correlations alone is not evidence of entanglement. Nonetheless, from a theoretical perspective there should be entanglement in the spin sector despite the fact that we observe relaxation dynamics between the different spin states involved. Since $g_{0,0}^{1, - 1} = g_{0,0}^{ - 1,1}$, $\hat H_{\mathrm{s}}$ only couples a pair initially in |0, 0〉 to the symmetrized states $\hat S\left| {1, - 1} \right\rangle$ and $\hat S\left| {2, - 2} \right\rangle$, which are both entangled two-atom spin states. The interaction does not provide coupling to antisymmetrized spin states, for example $\hat A\left| {1, - 1} \right\rangle \equiv \frac{1}{{\sqrt 2 }}\left( {\left| 1 \right\rangle _1 \otimes \left| { - 1} \right\rangle _2 - \left| { - 1} \right\rangle _1 \otimes \left| 1 \right\rangle _2} \right)$, since $\langle 1, - 1|\hat A^\dagger \hat H_{\mathrm{s}}|0,0\rangle = 0$. Consequently, the collisional interaction alone does not provide a route for relaxation into unentangled two-atom spin states such as |−1〉_1_ ⊗ |1〉_2_ or |1〉_1_ ⊗ |−1〉_2_, since these are superpositions of $\hat S\left| {1, - 1} \right\rangle$ and $\hat A\left| {1, - 1} \right\rangle$. The relaxation dynamics that we observe in the theoretical calculations, consistent with the experiment at low magnetic bias fields, is therefore a relaxation into a mixture of |0, 0〉, $\hat S\left| {1, - 1} \right\rangle$, and $\hat S\left| {2, - 2} \right\rangle$. Postselecting on any of the latter two entangled states therefore allows the preparation of a pure entangled state (see Supplementary Note 2).

Since |−1〉_1_ ⊗ |1〉_2_ is degenerate with |1〉_1_ ⊗ |−1〉_2_, unwanted effects such as magnetic field noise do not dephase $\hat S\left| {1, - 1} \right\rangle$ into a mixture of unentangled states. Other effects such as spin-orbit coupling and polarization gradients from the non-paraxial nature of the optical tweezer, which are not presently included in our modelling, might also affect the quality of the entangled state. However, the strong correlation we observe between *m*-state populations justifies our neglect of spin-orbit coupling, and our choice of large trap detuning and alignment of the bias magnetic field perpendicular to the fictitious magnetic field mitigate the effects of polarization variations. We therefore expect that it should be possible to observe long-lived entanglement generated by the collisional interaction. Since states of the form $\frac{1}{{\sqrt 2 }}\left( {\left| {1, - 1} \right\rangle + \left| { - 1,1} \right\rangle } \right)$ have applications to metrology and quantum information processing^45,46^, it is a future goal of ours to experimentally confirm the generation of the entangled state directly. For instance, exposing ${\textstyle{1 \over {\sqrt 2 }}}\,(|1, - 1\rangle + | - 1,1\rangle )$ to a $\frac{\pi }{2}$-pulse (effected by driving stimulated Raman transitions between the *m* = ±1 states) converts it to $- {\textstyle{i \over {\sqrt 2 }}}\,(|1,1\rangle + | - 1, - 1\rangle )$, which is identified by observing both atoms in the same *m*-state. If the entanglement was lost, we would observe both atoms in different *m*-states after the $\frac{\pi }{2}$-pulse with 50% probability.

In the context of observing entanglement in our atom-pair system, we make two remarks on the experimental data from the high bias magnetic field regime where the relaxation mechanism is not yet captured by our simulations. First, we observe strong correlations between the two atoms’ *m*-states in this regime, which is a requirement for entanglement. Secondly, Fig. 4 does not show relaxation to equal populations of all five spin states that conserve total magnetization (|0, 0〉, |−1〉_1_ ⊗ |1〉_2_, |1〉_1_ ⊗ |−1〉_2_, |−2〉_1_ ⊗ |2〉_2_, and |2〉_1_ ⊗ |−2〉_2_). Specifically, a *χ*-squared test reveals that the observed relative populations at the final time point in Fig. 4 significantly differ from $N_{m \, = \, 0} = \frac{1}{5}$ and $N_{m \, = \, \pm 1} = N_{m \, = \, \pm 2} = \frac{2}{5}$ (*χ*^2^ (*df* = 3) = 71.1, *p* < 0.001). In contrast, there is no statistically significant difference between these data and $N_{m \, = \, 0} = N_{m = \pm 1} = N_{m \, = \, \pm 2} = \frac{1}{3}(\chi ^2\left( {{\mathrm{df}} = 3} \right) = 1.9,p = 0.590)$. This could indicate that the antisymmetrized spin states remain unpopulated and entanglement is present in this regime. Although promising, these observations alone do not provide unequivocal evidence for entanglement in the spin sector.

To summarize, the bottom-up assembling of pairs from individual atoms allows us to study the collisional properties of ^85^Rb, whose effective attractive interactions are unfavourable for ultracold-ensemble collision experiments. A single pair of ^85^Rb atoms in an optical tweezer displays spin dynamics that yield strong correlation between magnetic substates of the two atoms. Unlike both finite-temperature many-body experiments and zero-temperature two-body experiments, our finite-temperature two-body experiments show relaxation dynamics rather than coherent spin waves. The record-high pair correlation measured is only limited by detection inefficiency; improving upon this technical limitation might allow studies of unexplored effects, such as violations of total magnetization conservation due to spin-orbit coupling, or studies of quantum relaxation processes and quantum thermodynamics. Our experiments indicate that spin-changing collisions may offer a useful finite-temperature entanglement resource that is robust to thermal noise.

## Methods

### Experimental procedure

We initially cool and trap a cloud of ^85^Rb atoms using magneto-optical trapping. We then load a small number of atoms from the cloud into two optical tweezers separated by ~4 μm, each with a trap width of ~1.05 μm and depth of *h* × 58 MHz. The two optical tweezers are formed by focusing two steerable linearly polarized laser beams (*λ* = 1064 nm) with a high-numerical-aperture lens (NA = 0.55). We use blue-detuned light-assisted collisions to reduce the occupancy of each trap to a single atom and confirm the presence of the two isolated atoms via fluorescence imaging^29,34,35^. The probability that there are two atoms, one in each tweezer, after the loading procedure is ~0.64, and we disregard the unsuccessful attempts.

After the loading process, the atoms are prepared in the desired *f* = 2, *m* = 0 groundstate in two steps. First, we optically pump atoms to the *f* = 3, *m* = 0 state by applying linearly polarized optical pumping light with two frequencies corresponding to the ^85^Rb *D*_1_ *f* = 2 to *f* ′ = 3 and the *f* = 3 to *f* ′ = 3 transitions. During this, the bias magnetic field of 8.5 Gauss defines the quantization axis for the atoms in the groundstate. This gives an atomic population of 0.99 occupying the *f* = 3, *m* = 0 state. Last, we apply a *π*-pulse (1.57 *μ*s) of co-propagating Raman beams (~36 GHz red detuned from the *D*_2_ line) to coherently transfer the atoms from the *f* = 3, *m* = 0 state to the *f* = 2, *m* = 0 state.

Using a 20 ms frequency sweep of an acousto-optical modulator, we adiabatically bring the two tweezers closer until they are merged (the distance between the centres of the two laser beams is ~900 nm). We then adiabatically ramp off one of the tweezers in ~17 ms while the other is simultaneously ramped to the desired trap depth and the bias magnetic field is set to the chosen value. The procedure leaves the atoms in the same optical tweezer where the collisional interactions generate the |*m*〉 population dynamics.

To observe the results shown in Figs. 2 and 4, we use the following experimental parameters: a trap depth of *h* × 58 MHz, oscillation frequencies 2*π* × 136 kHz and 2*π* × 22 kHz for the radial and axial dimensions, respectively, an atomic temperature of 107 μK, and a bias magnetic field of 8.5 Gauss. For Fig. 3, we use a trap depth of *h* × 10 MHz, oscillation frequencies 2*π* × 56 kHz and 2*π* × 9 kHz for the radial and axial dimensions, respectively, and an atomic temperature of 44 μK.

The detection of atoms in a particular |*m*〉 of the *f* = 2 manifold is done by ejecting the atoms out of the trap. In the presence of the magnetic field, we use a Raman process to transfer only the population in the specific |*m*〉 to the *f* = 3 manifold. We then deplete the *f* = 3 population using the push out technique^47^ and then measure the number of remaining atoms in the trap using fluorescence detection^48^. This procedure yields that the lost atoms were in the detected |*m*〉 while the remaining atoms were in the other states. In our push out technique, the detection efficiencies are 0.944 ± 0.004 and 0.997 ± 0.003 for the *f* = 2 and *f* = 3 states, respectively. In Fig. 4 the probability for |0, 0〉 $\left( {\hat S\left| {1, - 1} \right\rangle } \right)$ $\left[ {\hat S\left| {2, - 2} \right\rangle } \right]$ is determined by measuring the probability that zero atoms remain after atoms in the |0〉 (|1〉 and |−1〉) [|2〉 and |−2〉] are expelled.

### Relative number squeezing

The correlations between the |±1〉 of the two atoms (shown in Fig. 2) can be quantified by computing the population imbalance *J*_*z*_ = (*N*_+1_ − *N*_−1_)/2, and the relative number squeezing^16^ $\zeta ^2 = \frac{{\left( {\Delta J_z} \right)^2}}{{N/4}}$. Δ*J*_*z*_ is the standard deviation of *J*_*z*_, *N*_±1_ is number of atoms in |±1〉, and *N* is the total number of atoms. We deduce the number squeezing from the data in Fig. 2c at the collision times of 150, 250, 350, and 500 ms (see Supplementary Note 3 for values of *ζ*^ 2^ at these individual collision times). If we postselect on at least one atom being detected in |1〉 or |−1〉, the result of ejecting atoms from both |−1〉 and |1〉 have only two possible outcomes: (1) zero atoms remain in the tweezer, which indicates that one atom was in |−1〉 and another was in |1〉, and therefore *J*_*z*_ (*n* = 0) = 0; or (2) one atom remains after ejection, which indicates that one atom was in |±1〉 and the other was in |0〉, |−2〉 or |2〉, so consequently *J*_*z*_(*n* = 1) = ±0.5. Here, we assume that the probability of having both atoms in |1〉 or |−1〉 is zero.

Still restricting to the subspace where at least one atom is in |1〉 or |−1〉 and taking *P*_*n*_ to be the probability of *n* atoms remaining in the optical tweezer after ejection, we can determine that the mean population imbalance is zero:

2 $$\left\langle {J_z} \right\rangle = \frac{1}{{\left( {P_0 + P_1} \right)}}\mathop {\sum}\limits_{n = 0,1} {J_z} \left( n \right)P_n = \frac{{\left( {0 \times P_0 + 0.5\frac{{P_1}}{2} - 0.5\frac{{P_1}}{2}} \right)}}{{\left( {P_0 + P_1} \right)}} = 0.$$

The variance of the population imbalance, $({\mathrm{\Delta }}J_z)^2 = \left\langle {J_z^2} \right\rangle - \left\langle {J_z} \right\rangle ^2$, is given by:

3 $$(\Delta J_z)^2 = \left\langle {J_z^2} \right\rangle = \frac{1}{{\left( {P_0 + P_1} \right)}}\mathop {\sum}\limits_{n = 0,1} {\left( {J_z\left( n \right)} \right)^2} P_n = \frac{{0^2P_0 + 0.5^2P_1}}{{\left( {P_0 + P_1} \right)}}.$$

This allows us to quantify the degree of correlations between |1〉 and |−1〉 via the number squeezing parameter. From above, the number squeezing is given by

4 $$\zeta ^2 = \frac{{\left( {{\mathrm{\Delta }}J_z} \right)^2}}{{N/4}} = \frac{{P_1}}{{N\left( {P_0 + P_1} \right)}}.$$

Our measurement of the correlation can be influenced by the detection efficiency since the detection error in both *f* = 2 and *f* = 3 states will contribute to the measured value of *P*_1_. The directly measured variance (Δ*J*_*z*_)^2^ is 0.032 ± 0.002, while the detection error gives a variance of 0.034 ± 0.002 under the assumption that the actual (Δ*J*_*z*_)^2^ = 0. This shows the measured degree of relative number squeezing can be entirely attributed to the detection efficiency.

### Coupling strengths and rate equations

We deduce the transition rates from the spin-dependent interaction strengths. We assume that the transition rates between $\hat S|m, - m\rangle$ and $\hat S|m^\prime , - m^\prime \rangle$ are incoherent and have strengths proportional to $|\langle m^\prime , - m^\prime |\hat S\hat H_{\mathrm{s}}\hat S|m, - m\rangle |^2$. For low collisional energy, the interaction Hamiltonian of two atoms is approximated by^24^

5 $$\hat H_{\mathrm{s}} = V\left( {\hat {\mathbf{r}}} \right)\mathop {\sum}\limits_{m_1,m_2,m_1{\hskip -3pt \prime} ,m_2{\hskip -3pt \prime} } {g_{m_1,m_2}^{m_1{\hskip -3pt \prime} ,m_2{\hskip -3pt \prime} }} \left| {m_1{\hskip -3pt \prime} ,m_2{\hskip -3pt \prime} } \right\rangle \langle m_1,m_2|,$$

where $\hat {\mathbf{r}}$ is the relative position. The coupling coefficient between the initial |*m*_1_, *m*_2_〉 and final $\left| {m_1{\hskip -3pt \prime} ,m_2{\prime} } \right\rangle$ of the atom pair is

6 $$g_{m_1,m_2}^{m_1{\hskip -3pt \prime} ,m_2{\hskip -3pt \prime} } = \mathop {\sum}\limits_{F = 0}^{2f} {\mathop {\sum}\limits_{M = - F}^F {g_F} } \left\langle {m_1{\hskip -3pt \prime} ,m_2{\hskip -3pt \prime} |F,M} \right\rangle \left\langle {F,M|m_1,m_2} \right\rangle ,$$

where *g*_*F*_ = 4*π*ℏ^2^*a*_*F*_/*m* with *a*_*F*_ the *s*-wave scattering length for two atoms colliding in a channel with total spin *F*. As shown in Supplementary Note 4, provided both spin-2 atoms are initially prepared in the *m* = 0 Zeeman state, there are only six unique coupling coefficients in the above sum:

7 $$\begin{array}{*{20}{l}} {g_{0,0}^{0,0}} \hfill & = \hfill & {\frac{1}{{35}}\left( {7g_0 + 10g_2 + 18g_4} \right),} \hfill \\ {g_{0,0}^{1, - 1}} \hfill & = \hfill & {\frac{1}{{35}}\left( { - 7g_0 - 5g_2 + 12g_4} \right),} \hfill \\ {g_{0,0}^{2, - 2}} \hfill & = \hfill & {\frac{1}{{35}}\left( {7g_0 - 10g_2 + 3g_4} \right),} \hfill \\ {g_{1, - 1}^{1, - 1}} \hfill & = \hfill & {\frac{1}{{70}}\left( {14g_0 + 5g_2 + 16g_4} \right),} \hfill \\ {g_{1, - 1}^{2, - 2}} \hfill & = \hfill & {\frac{1}{{35}}\left( { - 7g_0 + 5g_2 + 2g_4} \right),} \hfill \\ {g_{2, - 2}^{2, - 2}} \hfill & = \hfill & {\frac{1}{{70}}\left( {14g_0 + 20g_2 + g_4} \right).} \hfill \end{array}$$

For ^85^Rb, the theoretically predicted *s*-wave scattering lengths are *a*_0_ = −740 ± 60 a.u., *a*_2_ = −570 ± 50 a.u., and *a*_4_ = −390 ± 20 a.u.^26^. By assuming the transition rate *γ*_*mm*′_ between $\hat S\left| {m, - m} \right\rangle$ and $\hat S\left| {m{\prime}, - m{\prime}} \right\rangle$ is proportional to $|\langle m{\prime}, - m{\prime}|\hat S\hat H_{\mathrm{s}}\hat S|m, - m\rangle |^2$ we get $\gamma _{01}/\gamma _{12} = \left( {\sqrt 2 g_{0,0}^{1, - 1}} \right)^2/\left( {2g_{1, - 1}^{2, - 2}} \right)^2 = 2.34 \pm 1.66$. Similarly, *γ*_02_/*γ*_01_ and *γ*_02_/*γ*_12_ equal $0.04_{ - 0.04}^{ + 0.08}$ and $0.09_{ - 0.09}^{ + 0.19}$, respectively. We therefore set *γ*_02_ to zero in the following rate equations.

Ignoring *γ*_02_, we use the following rate equation to model the experimental results in Fig. 4:

8 $$\begin{array}{*{20}{l}} {\frac{{dP_{\left| {0,0} \right\rangle }}}{{dt}}} \hfill & = \hfill & { - \gamma _{01}P_{\left| {0,0} \right\rangle } + \gamma _{01}P_{\hat S\left| {1, - 1} \right\rangle }} \hfill \\ {\frac{{dP_{\hat S\left| {1, - 1} \right\rangle }}}{{dt}}} \hfill & = \hfill & {\gamma _{01}P_{\left| {0,0} \right\rangle } - \left( {\gamma _{01} + \gamma _{12}} \right)P_{\hat S\left| {1, - 1} \right\rangle } + \gamma _{12}P_{\hat S\left| {2, - 2} \right\rangle }} \hfill \\ {\frac{{dP_{\hat S\left| {2, - 2} \right\rangle }}}{{dt}}} \hfill & = \hfill & {\gamma _{12}P_{\hat S\left| {1, - 1} \right\rangle } - \gamma _{12}P_{\hat S\left| {2, - 2} \right\rangle }} \hfill \end{array}$$

where $P_{\hat S\left| {m, - m} \right\rangle }$ is the $\hat S\left| {m, - m} \right\rangle$ population. Using the above ratio of rates, we set *γ*_01_ = 2.34 × *γ*_12_ and fit the entire experimental dataset in Fig. 4 using the single fitting parameter *γ*_12_.

### Theoretical model of collisional spin dynamics

We describe the collisional dynamics of two bosonic atoms in a three-dimensional anisotropic harmonic potential with Hamiltonian Eq. (1) and spin-changing interaction given by Eq. (5). As discussed above, since both *F* = 2 atoms are initially prepared in the *m* = 0 Zeeman state, binary collisions preserve the spin projection along the quantization axis. Consequently, only three two-particle spin states are accessible: |0, 0〉, $\hat S\left| {1, - 1} \right\rangle$, and $\hat S\left| {2, - 2} \right\rangle$. Writing the quantum state $|\psi (t)\rangle = \mathop {\sum}\limits_{m = 0,1,2} {{\int} d } {\mathbf{r}} \, \psi _m({\mathbf{r}},t)|{\mathbf{r}}\rangle \otimes \hat S|m, - m\rangle$, where ${\hat {\mathbf{r}}} |{\mathbf{r}}\rangle = {\mathbf{r}}|{\mathbf{r}}\rangle$, allows us to express the evolution under Hamiltonian (1) as

9 $$ \begin{array}{*{20}{l}} {i\hbar \dot \psi _0({\mathbf{r}})} \hfill & = \hfill & {H_{{\mathrm{rel}}}({\mathbf{r}})\psi _0({\mathbf{r}}) + V({\mathbf{r}})\left[ {g_{0,0}^{0,0}\psi _0({\mathbf{r}}) + \sqrt 2 g_{0,0}^{1, - 1}\psi _1({\mathbf{r}}) + \sqrt 2 g_{0,0}^{2, - 2}\psi _2({\mathbf{r}})} \right],} \hfill \\ {i\hbar \dot \psi _1({\mathbf{r}})} \hfill & = \hfill & {\left( {H_{{\mathrm{rel}}}({\mathbf{r}}) + \hbar q_1B^2} \right)\psi _1({\mathbf{r}}) + V({\mathbf{r}})\left[ {\sqrt 2 g_{0,0}^{1, - 1}\psi _0({\mathbf{r}}) + 2g_{1, - 1}^{1, - 1}\psi _1({\mathbf{r}}) + 2g_{1, - 1}^{2, - 2}\psi _2({\mathbf{r}})} \right],} \hfill \\ {i\hbar \dot \psi _2({\mathbf{r}})} \hfill & = \hfill & {\left( {H_{{\mathrm{rel}}}({\mathbf{r}}) + \hbar q_2B^2} \right)\psi _2(r) + V({\mathbf{r}})\left[ {\sqrt 2 g_{0,0}^{2, - 2}\psi _0({\mathbf{r}}) + 2g_{1, - 1}^{2, - 2}\psi _1({\mathbf{r}}) + 2g_{2, - 2}^{2, - 2}\psi _2({\mathbf{r}})} \right],} \hfill \end{array}$$

where $H_{{\mathrm{rel}}}({\mathbf{r}}) = - \frac{{\hbar ^2}}{{2\mu }}\nabla ^2{\mathbf{r}} + \frac{1}{2}\mathop {\sum}\nolimits_{i = x,y,z} \mu \omega _i^2r_i^2$, the coupling constants are given by Eq. (7), and the quadratic Zeeman shifts are *q*_1_ = 143.776 Hz/G^2^ and *q*_2_ = 575.104 Hz/G^2^^49^.

We take our initial condition as *ψ*_1_(**r**, 0) = *ψ*_2_(**r**, 0) = 0 and *ψ*_0_(**r**, 0) as a thermal distribution of even eigenstates of *H*_rel_(**r**). Specifically, in any given experiment $\psi _0({\mathbf{r}},0) = \varphi _{n_x}(x)\varphi _{n_y}(y)\varphi _{n_z}(z)$, where $\varphi _{n_i}(x_i)$ are eigenstates of the 1D harmonic oscillator with mass *μ* and frequency *ω*_*i*_ and $( - 1)^{n_x + n_y + n_z} = 1$ (since *ψ*_0_(**r**) must be symmetric under particle exchange). The Boltzmann probability that *ψ*_0_(**r**, 0) will be prepared in the eigenstate with quantum numbers (*n*_*x*_, *n*_*y*_, *n*_*z*_) is ${\cal{P}}(n_x,n_y,n_z) = \exp ( - \beta \varepsilon _{n_x,n_y,n_z})/{\cal{Z}}$, where $\varepsilon _{n_x,n_y,n_z} = \hbar \omega _x(n_x + {\textstyle{1 \over 2}}) + \hbar \omega _y(n_y + {\textstyle{1 \over 2}}) + \hbar \omega _z(n_z + {\textstyle{1 \over 2}})$ are the eigenstate energies, *β* = 1/*k*_B_*T*, and the partition function ${\cal{Z}}$ has an analytic expression (see Supplementary Note 5).

In the low-energy regime where *s*-wave collisions dominate, it is customary to take *V*(**r**) = *δ*(**r**)^24^. However, in this case spin-changing dynamics only occur for eigenstates where *n*_*x*_, *n*_*y*_, *n*_*z*_ are all even (see Supplementary Note 6). In contrast, states where (say) *n*_*x*_ is even and *n*_*y*_ and *n*_*z*_ are odd never evolve. These latter kinds of states represent roughly 70% of the ensemble at 44 *μ*K, implying that this model predicts that the population of |0, 0〉 never drops below 0.7, at odds with what we experimentally observe.

We wish to use a simplified atom-atom interaction model that allows for numerical calculations involving a high number of modes, while at the same time avoids the problem with the delta-function interaction model^50^. In particular, there is some evidence that the zero-range *δ*-function pseudopotential fails to replicate the scattering properties of the underlying physical potential in trapped systems when the magnitude of the *s*-wave scattering length is on the order or greater than the harmonic oscillator lengthscale^36,37^. Furthermore, there is a greater discrepancy for negative scattering lengths. In our experiment *a*_0_/*d* = −0.44, *a*_2_/*d* = −0.34, and *a*_4_/*d* = −0.23, where $d = \sqrt {\hbar /(m\bar \omega )}$ and $\bar \omega = (\omega _x\omega _y\omega _z)^{1/3}$. We use a Gaussian pseudopotential *V*(**r**) = exp[−*r*^2^/(2*w*^2^)]/(2*πw*^2^)^3/2^ with $w = \sqrt {(a_0^4 + a_2^4 + a_4^4)/(a_0^2 + a_2^2 + a_4^2)} \sim 650$ a.u., since (1) it is finite range and couples all even-parity eigenstates, (2) it gives the same total scattering cross section as the *δ*-function pseudopotential (see Supplementary Note 7), (3) it smoothly recovers the (regularized) *δ*-function in the *w* → 0 limit, and (4) the form of the spin-changing coupling matrix is sufficiently simple that a numeric calculation is tractable.

We numerically solve for the spin-changing dynamics by expanding *ψ*_*i*_(**r**) on a finite basis of even-parity eigenstates of $H_{{\mathrm{rel}}}({\mathbf{r}}):\psi _i({\mathbf{r}},t) = \mathop {\sum}\limits_{\varepsilon _{n_x,n_y,n_z} \le E_{{\mathrm{cut}}}} {c_{n_x,n_y,n_z}^i} (t)\varphi _{n_x}(x)\varphi _{n_y}(y)\varphi _{n_z}(z)\ ,$ where the sum is over all eigenstates with energy $\varepsilon _{n_x,n_y,n_z}$ less than some energy cutoff *E*_cut_. It is necessary to choose *E*_cut_ sufficiently large that $\mathop {\sum}\limits_{\varepsilon _{n_x,n_y,n_z} \le E_{{\mathrm{cut}}}} {\cal{P}} (n_x,n_y,n_z) \approx 1$ and coupling to the highest-energy, sparsely occupied modes is negligible. For the computational resources at our disposal, these conditions limit our calculations to temperatures no greater than 8.8 μK—roughly one fifth the temperature of the experiment.

In this basis the state is represented by **c** = [**c**^0^, **c**^1^, **c**^2^]^Τ^, where **c**^*i*^ is the vector of coefficients $c_{n_x,n_y,n_z}^i$ for modes satisfying $\varepsilon _{n_x,n_y,n_z} \le E_{{\mathrm{cut}}}$. Equations (9) imply $i \hbar \dot{\mathbf{c}}(t) = {\mathbf{H}} \, {\mathbf{c}}(t)$ with

10 $${\mathbf{H}} = \left( {\begin{array}{*{20}{c}} {\boldsymbol{\epsilon}} + g_{0,0}^{0,0}{\mathbf{T}} & {\sqrt 2 g_{0,0}^{1, - 1}{\mathbf{T}}} & {\sqrt 2 g_{0,0}^{2, - 2}{\mathbf{T}}} \\ {\sqrt 2 g_{0,0}^{2, - 2}{\mathbf{T}}} & {({\boldsymbol{\upepsilon}} + \hbar q_1B^2{\mathbf{I}}) + 2g_{1, - 1}^{1, - 1}{\mathbf{T}}} & {2g_{1, - 1}^{2, - 2}{\mathbf{T}}} \\ {\sqrt 2 g_{0,0}^{2, - 2}{\mathbf{T}}} & {2g_{1,1}^{2, - 2}{\mathbf{T}}} & {({\boldsymbol{\upepsilon}} + \hbar q_1B^2{\mathbf{I}}) + 2g_{2, - 2}^{2, - 2}{\mathbf{T}}} \end{array}} \right).$$

Here **ε** is a diagonal matrix with energies $\varepsilon _{n_x,n_y,n_z}$ along the diagonal and the coupling matrix **T** is defined via $T_{n_x,n_y,n_z}^{m_x,m_y,m_z} = {\cal{I}}_{n_x,m_x}{\cal{I}}_{n_y,m_y}{\cal{I}}_{n_z,m_z}/(2\pi w^2)^{3/2},$ where the integrals ${\cal{I}}_{n_i,m_i} = {\int} d x_i\varphi _{n_i}(x_i)\exp [ - x_i^2/(2w^2)]\varphi _{m_i}^i(x_i)$ have an analytic solution in terms of Gauss hypergeometric functions (see Supplementary Note 8). Diagonalizing ${\mathbf{H}} = {\mathbf{UDU}}^\dagger$ gives the solution ${\mathbf{c}}(t) = {\mathbf{U}}\exp [ - {\textstyle{i \over \hbar }}{\mathbf{D}}t]{\mathbf{U}}^\dagger {\mathbf{c}}(0)$. Thus, for a given initial condition $\psi _0({\mathbf{r}},0) = \varphi _{m_x}(x)\varphi _{m_y}(y)\varphi _{m_z}(z)$ we can compute the population of the *j*th two-boson spin state $N_{m_x,m_y,m_z}^j(t) = \mathop {\sum}\limits_{\varepsilon _{n_x,n_y,n_z} \le E_{{\mathrm{cut}}}} | c_{n_x,n_y,n_z}^j(t)|^2$. The total population of the *j*th two-boson spin state assuming a thermal initial state is given by an incoherent sum over $N_{m_x,m_y,m_z}^j(t)$ weighted by the Boltzmann probability ${\cal{P}}(m_x,m_y,m_z)$:

11 $$P_{|j, - j\rangle }(t) = \mathop {\sum}\limits_{\varepsilon _{m_x,m_y,m_z} \le E_{{\mathrm{cut}}}} {\cal{P}} (m_x,m_y,m_z)N_{m_x,m_y,m_z}^j(t).$$

This procedure was used to generate the simulation data plotted in Figs. 3–5.

## Supplementary information

Supplementary Info

## Data Availability

The source data underlying Figs. 2–5 and Supplementary Fig. 1 are provided as a Source Data file.

## Code availability

Simulation codes are available from Stuart S. Szigeti on reasonable request.
