# Supplementary material for: Thermally robust spin correlations between two 85Rb atoms in an optical microtrap
Source: Nat Commun. 2019 Apr 23;10:1889. doi: 10.1038/s41467-019-09420-6 (PMC6478867; doi:10.1038/s41467-019-09420-6)
Supplement: Supplementary file 1 — Supplementary Info [file 41467_2019_9420_MOESM1_ESM.pdf]

Thermally-robust spin correlations between two ^{85}Rb atoms in an optical microtrap: Supplementary Information

Sompet *et al.*

Thermally-robust spin correlations between two ^{85}Rb atoms in an optical microtrap: Supplementary Information

Pimonpan Sompet,[1] Stuart S. Szigeti,[1, 2] Eyal Schwartz,[1] Ashton S. Bradley,[1] and Mikkel F. Andersen[1]

[1] *The Dodd-Walls Centre for Photonic and Quantum Technologies, Department of Physics, University of Otago, Dunedin, New Zealand*

[2] *Department of Quantum Science, Research School of Physics and Engineering, The Australian National University, Canberra ACT 2601, Australia*

In this Supplementary Information we provide further details on (1) the $|\pm 2\rangle$ population dynamics, (2) the argument for the presence of thermally-robust, metrologically-useful entanglement in our atom-pair experiment, (3) the correlation between $|\pm 1\rangle$ at different collision times, (4) the spin-changing collisional coupling constants, (5) the analytic form for the initial thermal distribution used in our theoretical modelling, (6) why a δ-function pseudopotential is incapable of modelling the dynamics of our experiment, (7) the justification of the width chosen for our Gaussian pseudopotential, and (8) the coupling matrix that arises from a Gaussian pseudopotential.

Supplementary Note 1: $|\pm 2\rangle$ population dynamics

In addition to the m-state correlation results that we show in Fig. 2 of the main text, for $|0\rangle$ and $|\pm 1\rangle$, we do the same measurement for the case of $|\pm 2\rangle$. The results are shown in Supplementary Figure 1. Experimentally, the atom pair are initially prepared in $|0\rangle$. In Supplementary Figure 1a, after a given collision time, we eject atoms in $|-2\rangle$ from the trap. Since the probability of one atom remaining in the trap increases with the collision time, this indicates that the population of $|-2\rangle$ increases with collision time. In the case of ejecting both atoms from $|-2\rangle$ and $|+2\rangle$, only pair loss is observed, as shown in Supplementary Figure 1b. Combining Supplementary Figure 1a with Supplementary Figure 1b allows us to conclude that when one atom is in $|-2\rangle$, the other is in $|+2\rangle$. Therefore, the collisional dynamics result in correlations between the $|\pm 2\rangle$ populations. However, the correlations between the $|\pm 2\rangle$ populations are moderate compared to the measured $|\pm 1\rangle$ case presented in Fig. 2b and Fig. 2c in the main text. This could be due to imperfect π-pulse Raman transfer for the detection of atoms in $|\pm 2\rangle$ in the $F = 2$ manifold, which is more sensitive to magnetic noise compared to the $|\pm 1\rangle$ case.

Supplementary Note 2: Prospects of metrologically-useful entanglement generation

Here we provide a detailed theoretical argument showing that, in principle, our experiment is capable of generating thermally-robust entangled states which are metrologically useful.

Suppose we prepare a thermal ensemble of atom pairs in $|0, 0\rangle$ - i.e. $\hat{\rho}_0 = (\sum_{\mathbf{n}} \mathcal{P}(\mathbf{n})|\mathbf{n}\rangle\langle\mathbf{n}|) \otimes |0, 0\rangle\langle 0, 0|$, where $|\mathbf{n}\rangle \equiv |n_x, n_y, n_z\rangle$ denotes the even-parity eigenstates of the harmonic oscillator Hamiltonian and $\mathcal{P}(\mathbf{n})$ is a Boltzmann distribution. Then under Hamiltonian (1) of the main text this initial state will always evolve to a state of the form

$$\hat{\rho}(t) = \sum_{\mathbf{n}} \mathcal{P}(\mathbf{n})|\psi_{\mathbf{n}}(t)\rangle\langle\psi_{\mathbf{n}}(t)|, \tag{1}$$

where

$$|\psi_{\mathbf{n}}(t)\rangle = \sum_{\mathbf{m}} \left[c^0_{\mathbf{m}}(\mathbf{n}, t)|0, 0\rangle + c^1_{\mathbf{m}}(\mathbf{n}, t)\hat{\mathcal{S}}|1, -1\rangle + c^2_{\mathbf{m}}(\mathbf{n}, t)\hat{\mathcal{S}}|2, -2\rangle\right] \tag{2}$$

is the state that results from evolving the *pure* initial state $|\mathbf{n}\rangle$ under Hamiltonian (1) of the main text. The coefficients $c^j_{\mathbf{m}}(\mathbf{n}, t)$ are determined by the numerical procedure described in the Methods (for example, by diagonalising the matrix defined in Eq. (10) of the Methods).

We now show that tracing out the motional degrees of freedom of Eq. (1) results in a reduced density matrix with off-diagonal elements indicative of entanglement between two-particle spin states $|1, -1\rangle$ and $|-1, 1\rangle$ (and also between $|2, -2\rangle$ and $|-2, 2\rangle$). Explicitly, the reduced density matrix that only accounts for the spin degrees of freedom is

$$\hat{\rho}_{\mathrm{S}}(t) = \mathrm{Tr}_{\mathrm{M}}\left\{\hat{\rho}(t)\right\} = \sum_{\mathbf{n}} \mathcal{P}(\mathbf{n}) \sum_{\mathbf{m}} \langle\mathbf{m}|\psi_{\mathbf{n}}(t)\rangle\langle\psi_{\mathbf{n}}(t)|\mathbf{m}\rangle, \tag{3}$$

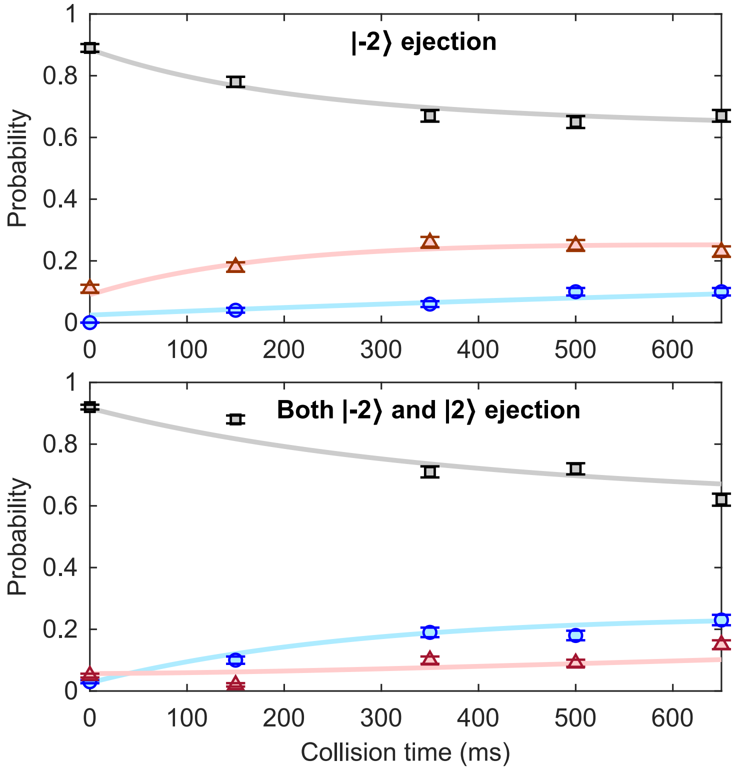

Supplementary Figure 1. **$|\pm 2\rangle$ population results.** Probability that zero, one, or two atoms remain in the optical tweezer after a given collision time. The error bars represent the standard error of the mean. **a,** When atoms solely from $|-2\rangle$ are expelled (immediately after a given collision time), this gives only single-atom loss events, which is opposite to the result in **b**. **b,** Expelling atoms from both $|-2\rangle$ and $|2\rangle$ gives only pair loss. In all cases and throughout the collision time, the bias magnetic field was 8.5 Gauss. The solid curves are a fit to the measured data, used to guide the eye. Source data are provided as a Source Data file.

where Tr_M denotes the partial trace over the motional degrees of freedom. Noting that

$$\langle \mathbf{m}|\psi_\mathbf{n}(t)\rangle = c^0_\mathbf{m}(\mathbf{n},t)|0,0\rangle + c^1_\mathbf{m}(\mathbf{n},t)\hat{\mathcal{S}}|1,-1\rangle + c^2_\mathbf{m}(\mathbf{n},t)\hat{\mathcal{S}}|2,-2\rangle, \tag{4}$$

and defining the coefficients

$$\rho_{i,j}(t) \equiv \sum_\mathbf{n} \mathcal{P}(\mathbf{n}) \sum_\mathbf{m} c^i_\mathbf{m}(\mathbf{n},t)[c^j_\mathbf{m}(\mathbf{n},t)]^*, \tag{5}$$

where $i,j = 0,1,2$, we can write $\hat{\rho}_\mathrm{S}(t)$ as

$$\begin{aligned}
\hat{\rho}_\mathrm{S}(t) = {} & \rho_{0,0}(t)|0,0\rangle\langle 0,0| + \tfrac{1}{2}\rho_{1,1}(t)\Big(|-1,1\rangle\langle -1,1| + |-1,1\rangle\langle 1,-1| + |1,-1\rangle\langle -1,1| + |1,-1\rangle\langle 1,-1|\Big) \\
& + \tfrac{1}{2}\rho_{2,2}(t)\Big(|-2,2\rangle\langle -2,2| + |-2,2\rangle\langle 2,-2| + |2,-2\rangle\langle -2,2| + |2,-2\rangle\langle 2,-2|\Big) \\
& + \Bigg[\tfrac{1}{\sqrt{2}}\rho_{0,1}(t)\Big(|0,0\rangle\langle -1,1| + |0,0\rangle\langle 1,-1|\Big) + \tfrac{1}{\sqrt{2}}\rho_{0,2}(t)\Big(|0,0\rangle\langle -2,2| + |0,0\rangle\langle 2,-2|\Big) \\
& \quad + \tfrac{1}{2}\rho_{1,2}(t)\Big(|-1,1\rangle\langle -2,2| + |-1,1\rangle\langle 2,-2| + |1,-1\rangle\langle 2,-2| + |1,-1\rangle\langle -2,2|\Big) + h.c.\Bigg],
\end{aligned} \tag{6}$$

or in matrix notation as

$$\hat{\rho}_{\mathrm{S}}(t) \equiv \begin{pmatrix} \rho_{0,0}(t) & \frac{1}{\sqrt{2}}\rho_{0,1}(t) & \frac{1}{\sqrt{2}}\rho_{0,1}(t) & \frac{1}{\sqrt{2}}\rho_{0,2}(t) & \frac{1}{\sqrt{2}}\rho_{0,2}(t) \\ \frac{1}{\sqrt{2}}\rho^*_{0,1}(t) & \frac{1}{2}\rho_{1,1}(t) & \frac{1}{2}\rho_{1,1}(t) & \frac{1}{2}\rho_{1,2}(t) & \frac{1}{2}\rho_{1,2}(t) \\ \frac{1}{\sqrt{2}}\rho^*_{0,1}(t) & \frac{1}{2}\rho_{1,1}(t) & \frac{1}{2}\rho_{1,1}(t) & \frac{1}{2}\rho_{1,2}(t) & \frac{1}{2}\rho_{1,2}(t) \\ \frac{1}{\sqrt{2}}\rho^*_{0,2}(t) & \frac{1}{2}\rho^*_{1,2}(t) & \frac{1}{2}\rho^*_{1,2}(t) & \frac{1}{2}\rho_{2,2}(t) & \frac{1}{2}\rho_{2,2}(t) \\ \frac{1}{\sqrt{2}}\rho^*_{0,2}(t) & \frac{1}{2}\rho^*_{1,2}(t) & \frac{1}{2}\rho^*_{1,2}(t) & \frac{1}{2}\rho_{2,2}(t) & \frac{1}{2}\rho_{2,2}(t) \end{pmatrix}. \tag{7}$$

Since $\rho_{1,1}(t)/2$ and $\rho_{2,2}(t)/2$ are just the populations in $|\pm 1, \mp 1\rangle$ and $|\pm 2, \mp 2\rangle$, respectively, we can clearly see that we are guaranteed entanglement between $|1,-1\rangle$ and $|-1,1\rangle$ (and similarly between $|2,-2\rangle$ and $|-2,2\rangle$) provided these populations are non-negligible. Indeed, via postselection we are guaranteed a maximally-entangled state.

Let us consider a concrete example, drawn upon the simulation data reported in the main text. For a relatively low magnetic bias field of $B = 0.2$ G and a temperature of 8.8 µK, after ~ 100 ms of evolution our simulation predicts a reduced density matrix of

$$\hat{\rho}_{\mathrm{S}}(t) = \begin{pmatrix} 0.5319 & 0.0002-0.0286i & 0.0002-0.0286i & 0.0115-0.0014i & 0.0115-0.0014i \\ 0.0002+0.0286i & 0.1477 & 0.1477 & -0.0023+0.0031i & -0.0023+0.0031i \\ 0.0002+0.0286i & 0.1477 & 0.1477 & -0.0023+0.0031i & -0.0023+0.0031i \\ 0.0115+0.0014i & -0.0023-0.0031i & -0.0023-0.0031i & 0.0846 & 0.0846 \\ 0.0115+0.0014i & -0.0023-0.0031i & -0.0023-0.0031i & 0.0846 & 0.0846 \end{pmatrix}. \tag{8}$$

In accordance with our intuition that spin-changing collisions preserve magnetization, the off-diagonal elements corresponding to entanglement between $|0,0\rangle$ and $|-1,1\rangle$, for instance, are much smaller than those corresponding to entanglement between $|1,-1\rangle$ and $|-1,1\rangle$. Indeed, if we postselect on the atoms being in the $m_F = \pm 1$ state, then 30% of the time we generate the maximally-entangled state $\frac{1}{\sqrt{2}}(|1,-1\rangle + |-1,1\rangle)$. We emphasize that this entanglement occurs for a thermal ensemble – that is, it is preserved under incoherent averaging over the motional degrees of freedom.

Furthermore, this entanglement is metrologically useful; as proven in Ref. [3], when a maximally-entangled state such as $\frac{1}{\sqrt{2}}(|1,-1\rangle + |-1,1\rangle)$ forms the input of a Ramsey or Mach-Zehnder interferometer it enables Heisenberg-limited sensitivities $\propto 1/N$. In contrast, the mixture $\frac{1}{2}\left(|1,-1\rangle\langle 1,-1| + |-1,1\rangle\langle -1,1|\right)$ allows metrology at sensitivities no better than the shot-noise limit $\propto 1/\sqrt{N}$. Both results follow from a computation of the quantum Fisher information, which is a necessary and sufficient witness of metrologically-useful multiparticle entanglement.

Experimentally, our observation of near-perfect pair correlations between magnetic sublevels is consistent with entanglement, but by itself is not a sufficient condition for entanglement. However, the observed relaxation of the spin populations to $N_{m_F=0} = N_{m_F=\pm 1} = N_{m_F=\pm 2} = 1/3$ is intriguing. Since atoms in different internal states can be considered distinguishable, complete incoherent thermalization between $|0,0\rangle$, $|-1,1\rangle$, $|1,-1\rangle$, $|-2,2\rangle$, and $|2,-2\rangle$ should result in $N_{m_F=0} = 1/5$ and $N_{m_F=\pm 1} = N_{m_F=\pm 2} = 2/5$. In contrast, if the symmetry of the atom-pair system only allows coupling between $|0,0\rangle$, $\hat{\mathcal{S}}|-1,1\rangle = \frac{1}{\sqrt{2}}(|1,-1\rangle + |-1,1\rangle)$, and $\hat{\mathcal{S}}|-2,2\rangle = \frac{1}{\sqrt{2}}(|2,-2\rangle + |-2,2\rangle)$, then relaxation within this subspace should yield equal populations of 1/3 – consistent with our experimental observations.

Supplementary Note 3: Pair correlation between $|+1\rangle$ and $|-1\rangle$ as a function of collision time

We quantify correlations between $|+1\rangle$ and $|-1\rangle$ by computing the relative number squeezing of the population imbalance, which is $\zeta^2 = P_1/[N(P_0 + P_1)]$ for our measurement (see Methods), where P_n is the probability of n atoms remaining in the optical tweezer after the ejection. In Supplementary Figure 2, the number squeezing is deduced from the data in Fig. 2c for different collision times (without correcting for detection inefficiency) in units of dB below quantum shot noise (QSN). The relative number fluctuations of 11.9 ± 0.3 dB below QSN stated in the main text is obtained by averaging over the measurement results at the collision times 150, 250, 350, and 500 ms.

Supplementary Note 4: Coupling coefficients for spin-exchange collision Hamiltonian

Consider the spin-changing interaction Hamiltonian

$$\hat{H}_{\mathrm{s}} = V(\hat{\mathbf{r}}) \sum_{m_1,m_2,m_3,m_4} g^{m_3,m_4}_{m_1,m_2} |m_3, m_4\rangle \langle m_1, m_2| , \tag{9}$$

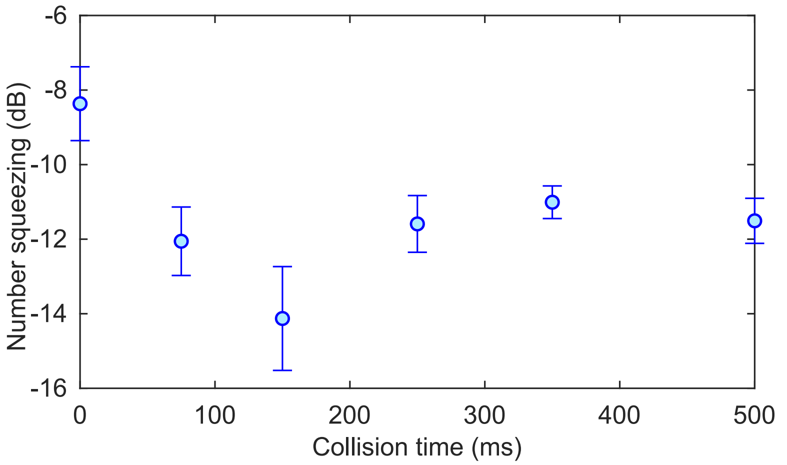

Supplementary Figure 2. **Number squeezing.** The relative number squeezing ζ^2 in units of dB below quantum shot noise (QSN) for different collision times, deduced from the data shown in Fig. 2c of the main text. The error bars denote the standard error of the mean ζ^2.

where

$$g_{m_1,m_2}^{m_3,m_4} = \sum_{F=0}^{2f} \sum_{M=-F}^{F} g_F \langle m_3, m_4 | F, M \rangle \langle F, M | m_1, m_2 \rangle \, , \tag{10}$$

and $g_F = 4\pi\hbar^2 a_F/m$ with a_F the s-wave scattering length for two atoms colliding in a channel with total spin F. Since both atoms are initially prepared in the $m = 0$ Zeeman state, and binary collisions conserve the spin projection along the quantization axis, the summation in Eq. (9) is highly constrained by $m_1 + m_2 = m_3 + m_4 = 0$. Here $|F, M\rangle$ are the eigenstates of the combined Hilbert space of two coupled spins, where F is the total angular momentum quantum number (for two spin-2 atoms, $F = 0, 2, 4$), and M the quantum number associated with the projection onto the quantization axis of this combined space. Consequently, $\langle m_3, m_4|F, M\rangle$ are Clebsch-Gordon coefficients. These can be taken to be real (so $\langle m_3, m_4|F, M\rangle = \langle F, M|m_3, m_4\rangle$), implying that

$$g_{m_1,m_2}^{m_3,m_4} = g_{m_3,m_4}^{m_1,m_2}. \tag{11}$$

Furthermore,

$$\langle m_1, m_2|F, M\rangle = (-1)^{2f-F} \langle m_2, m_1|F, M\rangle. \tag{12}$$

Since f and F are always even, $(-1)^{2f-F} = 1$ always, implying the symmetry

$$g_{m_1,m_2}^{m_3,m_4} = g_{m_2,m_1}^{m_3,m_4} = g_{m_1,m_2}^{m_4,m_3} = g_{m_2,m_1}^{m_4,m_3}. \tag{13}$$

Both permutation symmetries allow us to greatly simplify our expression for $\hat{H}_s$. For example,

$$g_{0,0}^{1,-1}|0,0\rangle\langle 1,-1| + g_{0,0}^{-1,1}|0,0\rangle\langle -1,1| = g_{0,0}^{1,-1}|0,0\rangle \left(\langle 1,-1| + \langle -1,1|\right) \equiv \sqrt{2} g_{0,0}^{1,-1}|0,0\rangle\langle 1,-1|\hat{S}, \tag{14}$$

and

$$\begin{aligned} &g_{1,-1}^{2,-2}|1,-1\rangle\langle 2,-2| + g_{-1,1}^{-2,2}|-1,1\rangle\langle -2,2| + g_{1,-1}^{-2,2}|1,-1\rangle\langle -2,2| + g_{-1,1}^{2,-2}|-1,1\rangle\langle 2,-2| \\ &= g_{1,-1}^{2,-2}\left(|1,-1\rangle + |-1,1\rangle\right)\left(\langle 2,-2| + \langle -2,2|\right) \\ &\equiv 2 g_{1,-1}^{2,-2}\hat{S}|1,-1\rangle\langle 2,2|\hat{S}, \end{aligned} \tag{15}$$

where $|0,0\rangle$, $\hat{S}\,|1,-1\rangle = \frac{1}{\sqrt{2}}\left(|1,-1\rangle + |-1,1\rangle\right)$, and $\hat{S}\,|2,-2\rangle = \frac{1}{\sqrt{2}}\left(|2,-2\rangle + |-2,2\rangle\right)$ are the only two-particle spin states accessible by our experiment, due to our choice of $m = 0$ initial condition.

These symmetries and simplifications allow us to write Hamiltonian (9) as

$$\begin{aligned}\hat{H}_{\rm s} = V(\hat{\mathbf{r}})\Big\{ & g_{0,0}^{0,0}|0,0\rangle\langle 0,0| + \sqrt{2}g_{0,0}^{1,-1}\left(|0,0\rangle\langle 1,-1|\hat{S} + \hat{S}|1,-1\rangle\langle 0,0|\hat{S}\right) + \sqrt{2}g_{0,0}^{2,-2}\left(|0,0\rangle\langle 2,-2|\hat{S} + \hat{S}|2,-2\rangle\langle 0,0|\right) \\ & + 2g_{1,-1}^{1,-1}\hat{S}|1,-1\rangle\langle 1,-1|\hat{S} + 2g_{2,-2}^{2,-2}\hat{S}|2,-2\rangle\langle 2,-2|\hat{S} + 2g_{1,-1}^{2,-2}\left(\hat{S}|1,-1\rangle\langle 2,-2|\hat{S} + \hat{S}|2,-2\rangle\langle 1,-1|\hat{S}\right)\Big\} \\ = V(\hat{\mathbf{r}}) \times & \begin{pmatrix} g_{0,0}^{0,0} & \sqrt{2}g_{0,0}^{1,-1} & \sqrt{2}g_{0,0}^{2,-2} \\ \sqrt{2}g_{0,0}^{1,-1} & 2g_{1,-1}^{1,-1} & 2g_{1,-1}^{2,-2} \\ \sqrt{2}g_{0,0}^{2,-2} & 2g_{1,-1}^{2,-2} & 2g_{2,-2}^{2,-2} \end{pmatrix}. \end{aligned} \tag{16}$$

Clearly the spin-changing interaction Hamiltonian is determined by six unique coupling coefficients. It is straightforward to compute these coupling coefficients; since $\langle F, M|m_1; m_2\rangle = 0$ for $m_1 + m_2 \neq M$, we only need to keep terms in Eq. (10) where $M = 0$:

$$g_{m_1,m_2}^{m_3,m_4} = g_0\langle m_3, m_4|0,0\rangle\langle 0,0|m_1, m_2\rangle + g_2\langle m_3, m_4|2,0\rangle\langle 2,0|m_1, m_2\rangle + g_4\langle m_3, m_4|4,0\rangle\langle 4,0|m_1, m_2\rangle. \tag{17}$$

Explicitly evaluating the Clebsch-Gordon coefficients gives Eqs. (7) in the Methods.

Supplementary Note 5: Thermal distribution for two-particle states in a 3D harmonic potential

Initially, our two atoms are always in the $|0,0\rangle$ state, so $\psi_1(\mathbf{r}, 0) = \psi_2(\mathbf{r}, 0) = 0$ (recall that $\langle \mathbf{r}|\psi(t)\rangle = \sum_{m=0,1,2}\psi_m(\mathbf{r}, t)\hat{S}|m, -m\rangle$). In any given experiment, the two atoms are prepared in a specific eigenstate $\psi_0(\mathbf{r}, 0) = \varphi_{n_x}(x)\varphi_{n_y}(y)\varphi_{n_z}(z)$, but *only* for values of n_x, n_y, n_z where $(-1)^{n_x+n_y+n_z} = 1$ (since $\psi_0(\mathbf{r})$ must be symmetric under particle exchange). Here $\varphi_{n_i}(x_i)$ are the eigenstates of the 1D harmonic oscillator of mass μ and frequency ω_i.

Assuming these constraints on the allowable eigenstates, then within the canonical ensemble (i.e. Boltzmann statistics), the probability that $\psi_0(\mathbf{r}, 0)$ will be prepared in the eigenstate with quantum numbers (n_x, n_y, n_z) is

$$\mathcal{P}(n_x, n_y, n_z) = \frac{1}{\mathcal{Z}}\exp\left\{-\beta\left[\hbar\omega_x(n_x + \tfrac{1}{2}) + \hbar\omega_y(n_y + \tfrac{1}{2}) + \hbar\omega_z(n_z + \tfrac{1}{2})\right]\right\}, \tag{18}$$

where $\beta = 1/k_{\rm B}T$ and $\mathcal{Z}$ is the partition function, given by the sum over Boltzmann factors for each *allowable* state. This can be written

$$\mathcal{Z} = \mathcal{Z}_x^{\rm even}\mathcal{Z}_y^{\rm even}\mathcal{Z}_z^{\rm even} + \mathcal{Z}_x^{\rm odd}\mathcal{Z}_y^{\rm odd}\mathcal{Z}_z^{\rm even} + \mathcal{Z}_x^{\rm odd}\mathcal{Z}_y^{\rm even}\mathcal{Z}_z^{\rm odd} + \mathcal{Z}_x^{\rm even}\mathcal{Z}_y^{\rm odd}\mathcal{Z}_z^{\rm odd}, \tag{19}$$

where

$$\mathcal{Z}_i^{\rm even} = \sum_{m_i=0}^{\infty} e^{-\beta\hbar\omega_i(2m_i+1/2)}, \tag{20}$$

$$\mathcal{Z}_i^{\rm odd} = \sum_{m_i=0}^{\infty} e^{-\beta\hbar\omega_i[(2m_i+1)+1/2]}, \tag{21}$$

are the partition functions corresponding to the even and odd states of a 1D harmomic oscillator of frequency ω_i, respectively. Analytic expressions for these partition functions exist. For the even case, first note that

$$\mathcal{Z}_i^{\rm even} = q_i^{1/4}\left(1 + q_i + q_i^2 + \cdots\right), \tag{22}$$

where we have defined $q_i \equiv \exp(-2\beta\hbar\omega_i)$. Clearly $q_i\mathcal{Z}_i^{\rm even} = q_i^{1/4}\left(q_i + q_i^2 + q_i^3 + \cdots\right)$, so $\mathcal{Z}_i^{\rm even} - q_i Z_i^{\rm even} = q_i^{1/4}$, and therefore

$$\mathcal{Z}_i^{\rm even} = \frac{q_i^{1/4}}{1 - q_i} = \frac{e^{-\beta\hbar\omega_i/2}}{1 - e^{-2\beta\hbar\omega_i}}. \tag{23}$$

Similarly, we can show that

$$\mathcal{Z}_i^{\rm odd} = q_i^{3/4}\left(1 + q_i + q_i^2 + \cdots\right) = \frac{q_i^{3/4}}{1 - q_i} = \frac{e^{-3\beta\hbar\omega_i/2}}{1 - e^{-2\beta\hbar\omega_i}}. \tag{24}$$

Supplementary Note 6: Spin-changing collisions for δ-function potential

Here we derive the relative motion spin-changing evolution equations assuming a δ-function scattering interaction: $V(\mathbf{r}) = \delta(\mathbf{r})$. We expand $\psi_i(\mathbf{r}, t)$ in a basis of eigenstates of $H_{\text{rel}}(\mathbf{r}) = -\frac{\hbar^2}{2\mu}\nabla^2_{\mathbf{r}} + \frac{1}{2}\sum_{i=x,y,z}\mu\omega_i^2 r_i^2$:

$$\psi_i(\mathbf{r}, t) = \sum_{\mathbf{n}\in\mathcal{C}} c^i_{\mathbf{n}}(t)\phi_{\mathbf{n}}(\mathbf{r}), \tag{25}$$

where $\mathbf{n} = (n_x, n_y, n_z)$, $\phi_{\mathbf{n}}(\mathbf{r}) = \varphi_{n_x}(x)\varphi_{n_y}(y)\varphi_{n_z}(z)$, $\mathcal{C} = \{\mathbf{n} : \epsilon_{\mathbf{n}} \leq E_{\text{cut}}\}$, and $\epsilon_{\mathbf{n}} = \hbar\omega_x(n_x + 1/2) + \hbar\omega_y(n_y + 1/2) + \hbar\omega_z(n_x + 1/2)$. That is, we only consider a finite number of modes below some energy cutoff E_{cut}. The 1D Hermite-Gauss modes $\varphi_{n_i}(x_i)$ satisfy

$$\left[-\frac{\hbar^2}{2\mu}\frac{\partial^2}{\partial x_i} + \frac{1}{2}\mu\omega_i^2 x_i^2\right]\varphi_{n_i}(x_i) = \hbar\omega_i(n_i + \tfrac{1}{2})\varphi_{n_i}(x_i), \tag{26}$$

and are explicitly given by

$$\varphi_{n_i}(x_i) = (\sigma_i^2\pi)^{-1/4}\frac{1}{\sqrt{2^{n_i}n_i!}}H_{n_i}(x_i/\sigma_i)e^{-(x_i/\sigma_i)^2/2}, \tag{27}$$

where $\sigma_i = \sqrt{\hbar/(\mu\omega_i)}$, and $H_n(x)$ are Hermite polynomials.

Substituting Eq. (25) into Eqs (9) from the Methods, multiplying both sides by $\phi^*_{\mathbf{m}}(\mathbf{r})$, integrating over space, and then exploiting the orthonormality of the eigenstates, we obtain

$$i\hbar\dot{c}^0_{\mathbf{n}} = \epsilon_{\mathbf{n}}c^0_{\mathbf{n}} + \phi_{\mathbf{n}}(0)\sum_{\mathbf{m}}\phi_{\mathbf{m}}(0)\left[g^{0,0}_{0,0}c^0_{\mathbf{m}} + \sqrt{2}g^{1,-1}_{0,0}c^1_{\mathbf{m}} + \sqrt{2}g^{2,-2}_{0,0}c^2_{\mathbf{m}}\right], \tag{28a}$$

$$i\hbar\dot{c}^1_{\mathbf{n}} = \left(\epsilon_{\mathbf{n}} + \hbar q_1 B^2\right)c^1_{\mathbf{n}} + \phi_{\mathbf{n}}(0)\sum_{\mathbf{m}}\phi_{\mathbf{m}}(0)\left[\sqrt{2}g^{1,-1}_{0,0}c^0_{\mathbf{m}} + 2g^{1,-1}_{1,-1}c^1_{\mathbf{m}} + 2g^{2,-2}_{1,-1}c^2_{\mathbf{m}}\right], \tag{28b}$$

$$i\hbar\dot{c}^2_{\mathbf{n}} = \left(\epsilon_{\mathbf{n}} + \hbar q_2 B^2\right)c^2_{\mathbf{n}} + \phi_{\mathbf{n}}(0)\sum_{\mathbf{m}}\phi_{\mathbf{m}}(0)\left[\sqrt{2}g^{2,-2}_{0,0}c^0_{\mathbf{m}} + 2g^{2,-2}_{1,-1}c^1_{\mathbf{m}} + 2g^{2,-2}_{2,-2}c^2_{\mathbf{m}}\right], \tag{28c}$$

where $\phi_{\mathbf{n}}(0) = \varphi_{n_x}(0)\varphi_{n_y}(0)\varphi_{n_z}(0)$ is given by the simple expression

$$\varphi^i_{n_i}(0) = \begin{cases}\sigma_i^{-1/2}\frac{(-2)^{n_i/2}\sqrt{n_i!}}{\pi^{1/4}(n_i/2)!}, & n_i \text{ even},\\ 0, & n_i \text{ odd}.\end{cases} \tag{29}$$

Equation (29) implies that $\phi_{\mathbf{n}}(0)$ is only nonzero if n_x, n_y, and n_z are all even. Therefore, if the two-particle wavefunction for the $|0,0\rangle$ spin state is initially prepared in eigenstate $\phi_{\mathbf{n}}(\mathbf{r})$, then coupling to (symmetrized) spin states $\hat{S}|1,-1\rangle$, and $\hat{S}|2,-2\rangle$ only occurs if n_x, n_y, and n_z are all even. However, even-parity states where, for example, n_x is even and n_y and n_z are odd do not undergo spin-changing collisional dynamics according to this model. For our experiment, this represents a significant fraction of the thermal ensemble: for experimental parameters $T = 44\,\mu\text{K}$, $\omega_x = 2\pi\times 8.9$ kHz, $\omega_y = 2\pi\times 55.5$ kHz, and $\omega_z = 1.01\omega_y$, we have

$$\begin{aligned}\mathcal{F} &= \text{Fraction of states that do not change spin states under } \delta\text{-function potential}\\ &= \frac{\mathcal{Z}^{\text{odd}}_x\mathcal{Z}^{\text{odd}}_y\mathcal{Z}^{\text{even}}_z + \mathcal{Z}^{\text{odd}}_x\mathcal{Z}^{\text{even}}_y\mathcal{Z}^{\text{odd}}_z + \mathcal{Z}^{\text{even}}_x\mathcal{Z}^{\text{odd}}_y\mathcal{Z}^{\text{odd}}_z}{\mathcal{Z}}\\ &\approx 0.733.\end{aligned} \tag{30}$$

This model is therefore at odds with our experimental observations, which showed spin-changing collisional dynamics leading to a transfer of much more than 70% of the $|0,0\rangle$ population to the two-particle spin states $|\pm1,\mp1\rangle$ and $|\pm2,\mp2\rangle$.

We briefly remark that this conclusion remains true when the regularized δ-function potential is used: $V(\mathbf{r}) = \delta_{\text{reg}}(\mathbf{r}) \equiv \delta(\mathbf{r})\partial_r r = \delta(\mathbf{r})\left(1 + x\partial_x + y\partial_y + z\partial_z\right)$, where $\partial_{x_i} \equiv \partial/\partial x_i$.

Supplementary Note 7: Width of Gaussian scattering potential

Our numerical simulations use a normalized Gaussian scattering pseudopotential $V(\mathbf{r}) = \exp(-r^2/2w^2)/(2\pi w^2)^{3/2}$ with width $w^2 = (a_0^4 + a_2^4 + a_4^4)/(a_0^2 + a_2^2 + a_4^2)$, where the a_F are the s-wave scattering lengths for each total spin-F state. Here we show that this choice of width w gives the same overall low-energy scattering cross section (in free space) as the δ-function potential.

Within the Born approximation, the cross section for the spin-F channel is given by [1]

$$\begin{aligned}\frac{d\sigma_F}{d\Omega} &= \frac{\mu^2}{\hbar^4 q^2}\left|\int d\mathbf{r}e^{-i\mathbf{q}\cdot\mathbf{r}}V_F(\mathbf{r}) + \int d\mathbf{r}e^{i\mathbf{q}\cdot\mathbf{r}}V_F(\mathbf{r})\right|^2 \\ &= \frac{\mu^2}{\hbar^4 q^2}\left|\frac{4\pi}{q}\int_0^\infty dr\, r\sin(qr)V_F(r) + \frac{4\pi}{(-q)}\int_0^\infty dr\, r\sin(-qr)V_F(r)\right|^2 \\ &= \frac{16\mu^2}{\hbar^4 q^2}\left|\int_0^\infty dr\, r\sin(qr)V_F(r)\right|^2 . \end{aligned} \tag{31}$$

Here $V_F(\mathbf{r}) = g_F V(\mathbf{r})$, $\mathbf{q} \equiv \mathbf{k} - \mathbf{k}_0$ and $q = |\mathbf{q}|$, where $\mathbf{k}$ and $\mathbf{k}_0$ represent the momenta of incoming and outgoing plane waves (before and after scattering, respectively). The second term within the absolute value arises due to our requirement that for bosonic particles, the wavefunction needs to be symmetrized; this term is the same as the first but with $\mathbf{q} \to -\mathbf{q}$ (i.e. we are enforcing exchange symmetry), and for our radially-symmetric potential only results in an additional factor of 4 out the front. Since

$$\int_0^\infty dr\, r\sin(qr)V_F(r) = \frac{g_F}{4\pi}qe^{-w^2q^2/2}, \tag{32}$$

$g_F = 4\pi\hbar^2 a_F/m$, and $\mu = m/2$, we can write

$$\frac{d\sigma_F}{d\Omega} = 4a_F^2 e^{-w^2q^2}. \tag{33}$$

For a radially-symmetric potential, conservation of energy implies that $|\mathbf{k}| = |\mathbf{k}_0| \equiv k$. We can therefore write q in terms of k and the angle, θ, between $\mathbf{k}$ and $\mathbf{k}_0$: $q = 2k\sin(\theta/2)$.

To determine σ_F, we need to integrate over all *distinct* final scattering states, parametrized by the solid angle Ω. We must therefore only integrate from $0 \le \theta \le \pi/2$, $0 \le \phi \le 2\pi$, since the other half-shell $\pi/2 < \theta \le \pi$ is an identical set of scattered states (follows from the symmetrization requirement; scattering is invariant under exchange $\mathbf{q} \to -\mathbf{q}$, or $\theta \to \pi - \theta$). Thus,

$$\begin{aligned}\sigma_F(k) &= 4a_F^2 \times \underbrace{2\pi}_{\phi\text{ integral}} \times \int_0^{\pi/2} d\theta\, \sin\theta e^{-4w^2k^2\sin^2(\theta/2)} \\ &= 8\pi a_F^2\left(\frac{1 - e^{-2w^2k^2}}{2k^2w^2}\right) \\ &\approx 8\pi a_F^2\left(1 - w^2k^2\right), \end{aligned} \tag{34}$$

where the final line is approximately true in the limit of low-energy scattering. The *total* cross section is given by the sum over all spin-F channels:

$$\sigma_{\text{tot}}(k) = \sum_F 8\pi a_F^2\left(\frac{1 - e^{-2w^2k^2}}{2k^2w^2}\right) \approx 8\pi\left[\left(\sum_F a_F^2\right) - \left(\sum_F a_F^2\right)w^2k^2\right]. \tag{35}$$

Now compare this to the cross section for the δ-function pseudopotential [1]:

$$\sigma_F(k) = \frac{8\pi a_F^2}{1 + k^2a_F^2} \approx 8\pi a_F^2\left(1 - a_F^2k^2\right), \tag{36}$$

and so

$$\sigma_{\text{tot}}(k) \approx \sum_F 8\pi a_F^2\left(1 - a_F^2k^2\right) = 8\pi\left[\left(\sum_F a_F^2\right) - \left(\sum_F a_F^4\right)k^2\right]. \tag{37}$$

We therefore match the total cross section in the low-energy regime by choosing the width of our Gaussian as:

$$w^2 = \frac{\sum_F a_F^4}{\sum_F a_F^2} = \frac{a_0^4 + a_2^4 + a_4^4}{a_0^2 + a_2^2 + a_4^2}. \tag{38}$$

Supplementary Note 8: Coupling matrix for Gaussian pseudopotential

As can be seen from Hamiltonian Eq. (10) in the Methods, for the Gaussian pseudopotential, coupling to different spin states is described by the coupling matrix

$$[\mathbf{T}]_{\mathbf{n},\mathbf{m}} = \frac{1}{(2\pi w^2)^{3/2}} \mathcal{I}_{n_x,m_x} \mathcal{I}_{n_y,m_y} \mathcal{I}_{n_z,m_z}, \tag{39}$$

where

$$\begin{aligned}
\mathcal{I}_{n_i,m_i} &= \int dx_i\, \varphi_{n_i}(x_i) e^{-x_i^2/2w^2} \varphi_{m_i}(x_i) \\
&= \left(\pi 2^{n_i+m_i} n_i! m_i!\right)^{-1/2} \sigma_i^{-1} \int dx_i H_{n_i}(x_i/\sigma_i) H_{m_i}(x_i/\sigma_i) e^{-\left(1+\frac{\sigma_i^2}{2w^2}\right)\left(\frac{x_i}{\sigma_i}\right)^2} \\
&= \left(\pi 2^{n_i+m_i} n_i! m_i!\right)^{-1/2} \int d\tilde{x}_i H_{n_i}(\tilde{x}_i) H_{m_i}(\tilde{x}_i) e^{-2\alpha_i^2 \tilde{x}_i^2},
\end{aligned} \tag{40}$$

where $2\alpha_i^2 \equiv 1 + \sigma_i^2/(2w^2)$ and $\sigma_i = \sqrt{\hbar/(\mu\omega_i)}$. From result 7.374.5 of Ref. [2],

$$\int d\tilde{x} H_n(\tilde{x}) H_m(\tilde{x}) e^{-2\alpha^2 \tilde{x}^2} = 2^{\frac{m+n-1}{2}} \alpha^{-m-n-1} (1-2\alpha^2)^{\frac{m+2}{2}} \Gamma\left(\tfrac{m+n+1}{2}\right) F\left(-m, -n; \tfrac{1-m-n}{2}; \tfrac{\alpha^2}{2\alpha^2-1}\right), \tag{41}$$

if $m+n$ is even. If $m+n$ is odd, then this integral is zero. Here $F(a,b;c;d)$ is a Gauss hypergeometric function. Then

$$\mathcal{I}_{n_i,m_i} = \begin{cases} \frac{\Gamma\left(\frac{m_i+n_i+1}{2}\right)}{\sqrt{2\pi n_i! m_i!}} \alpha_i^{-m_i-n_i-1} (1-2\alpha_i^2)^{\frac{m_i+n_i}{2}} F\left(-m_i, -n_i; \tfrac{1-m_i-n_i}{2}; \tfrac{\alpha_i^2}{2\alpha_i^2-1}\right), & n_i + m_i \text{ even} \\ 0, & n_i + m_i \text{ odd.} \end{cases} \tag{42}$$

[1] Dalibard, J. *Collisional dynamics of ultracold atomic gases*, in Bose-Einstein Condensation in Atomic Gases 1998 321 - 349, (Course CXL of Proceedings of the International School of Physics "Enrico Fermi", IOS Press, 1998).

[2] Gradshteyn I. S. & Ryzhik, I. M. *Table of Integrals, Series, and Products, 8th edition.* Academic Press (2014).

[3] Pezzé L. & Smerzi, A. Entanglement, Nonlinear Dynamics, and the Heisenberg Limit. *Phys. Rev. Lett.* **102**, 100401 (2009).
